# Alteration of Intestinal Microbiota in 3-Deoxyglucosone-Induced Prediabetic Rats

**DOI:** 10.1155/2020/8406846

**Published:** 2020-08-25

**Authors:** Jin Cai, Liang Zhou, Xiudao Song, Meiqi Yin, Guoqiang Liang, Heng Xu, Lurong Zhang, Guorong Jiang, Fei Huang

**Affiliations:** ^1^Clinical Pharmaceutical Laboratory of Traditional Chinese Medicine, Suzhou TCM Hospital Affiliated to Nanjing University of Chinese Medicine, Suzhou, 215009 Jiangsu, China; ^2^Clinical Pharmaceutical Laboratory of Traditional Chinese Medicine, Suzhou Academy of Wumen Chinese Medicine, Suzhou, 215009 Jiangsu, China; ^3^Nanjing University of Chinese Medicine, Nanjing, 210023 Jiangsu, China; ^4^Department of Endocrinology, Suzhou TCM Hospital Affiliated to Nanjing University of Chinese Medicine, Suzhou, 215009 Jiangsu, China

## Abstract

Our previous research suggests that 3-deoxyglucosone (3DG), formed in the caramelization course and Maillard reactions in food, is an independent factor for the development of prediabetes. Since the relationship between type 2 diabetes (T2D) and intestinal microbiota is moving from correlation to causality, we investigated the alterations in the composition and function of the intestinal microbiota in 3DG-induced prediabetic rats. Rats were given 50 mg/kg 3DG by intragastric administration for two weeks. Microbial profiling in faeces samples was determined through the 16S rRNA gene sequence. The glucagon-like peptide 2 (GLP-2) and lipopolysaccharide (LPS) levels in plasma and intestinal tissues were measured by ELISA and Limulus test, respectively. 3DG treatment did not significantly change the richness and evenness but affected the composition of intestinal microbiota. At the phylum level, 3DG treatment increased the abundance of nondominant bacteria *Proteobacteria* but did not cause the change of the dominant bacteria. Meanwhile, the abundance of the *Prevotellaceae* family and *Parasutterela* genus and the *Alcaligencaeae* family and *Burkholderiales* order and its attachment to the *Betaproteobacteria* class were overrepresented in the 3DG group. The bacteria of *Candidatus Soleaferrea* genus, *Gelria* genus, and *Thermoanaerobacteraceae* family and its attachment to *Thermoanaerobacterales* order were apparently more abundant in the control group. In addition, 45 KEGG pathways were altered after two-week intragastric administration of 3DG. Among these KEGG pathways, 13 KEGG pathways were involved in host metabolic function related to amino acid metabolism, carbohydrate metabolism, metabolism of cofactors and vitamins, and metabolism of terpenoids and polyketides. Moreover, the increased LPS levels and the decreased GLP-2 concentration in plasma and intestinal tissues were observed in 3DG-treated rats, together with the impaired fasting glucose and oral glucose tolerance. The alterations in composition and function of the intestinal microbiota were observed in 3DG-treated rats, which provides a possible mechanism linking exogenous 3DG intake to the development of prediabetes.

## 1. Introduction

The changes in the dietary pattern structure have been shown to be related to the development of glucose metabolism diseases. It is well known that an increase in dietary fat and carbohydrate intake is an important risk factor for glucose homeostasis disorders [1, 2]. However, after the effective control of high-glucose and high-fat diet, the incidence of glucose metabolic disease does not significantly decrease, suggesting that there are other important factors in our diet involved in the occurrence and development of glucose homeostasis disorders. Accumulating evidence indicates that 1,2-dicarbonyl compounds, easily formed in the caramelization process and Maillard reaction in food, increase the risk of type 2 diabetes mellitus and its complications [3, 4]. Through the investigation of the content of 1,2-dicarbonyl compounds in food ingested daily, it is indicated that 3-deoxyglucosone (3DG) is the predominant 2-dicarbonyl compound [5]. Fruit juices, balsamic vinegar, honey, and bakery products such as cookies contain high levels of 3DG [5]. Several lines of evidence indicated that the plasma levels of 3DG in diabetic patients are increased [6–8]. In our previous clinical study, the elevation of plasma 3DG in nondiabetic seniors may lead to impaired glucose regulation (prediabetes) [9]. Emerging evidence by our group in animal studies further indicates that 3DG is an independent factor associated with the development of prediabetes [10–13]. An earlier study showed that the absorption rate of 3DG is low [14], suggesting that 3DG may mainly act on the gut. Indeed, our *in vivo* studies in rats have demonstrated that accumulation of 3DG in intestinal tissue induced by exogenous 3DG (50 mg/kg) impaired intestinal L-cell function [10] and gut permeability [15].

Intestinal microbiota has been becoming a hot topic, and accumulating studies indicate that it is an important organ involved in maintaining metabolic homeostasis. The relationship between the change of intestinal microbiota environment and the occurrence of metabolic diseases such as obesity and diabetes is moving from correlation to causation [16]. Intestinal microbiota disorder, such as the decrease of beneficial bacteria and the increase of harmful bacteria, is easy to occur in prediabetes and type 2 diabetes (T2D) patients [17]. Specific changes in intestinal microbiota are considered to be one of the causes of metabolic diseases [16, 18]. Diet is the most important factor in regulating intestinal microbiota. Changes in the dietary structure lead to the occurrence and development of metabolic diseases by affecting the composition and function of intestinal microbiota. For example, alterations in intestinal microbiota inhibit the transformation of bile acid in the lipid and glucose metabolism disturbance induced by a high-fat diet [19].

We conducted a study in 3DG-induced prediabetic rats to investigate the alterations in the composition and function of the intestinal microbiota and whether the effects of intestinal microbiota were associated with intestinal mucosal barrier dysfunction.

## 2. Materials and Methods

### 2.1. Synthesis of 3DG

3DG was synthesized from glucose as previously described according to the method of Wang et al. [20, 21].

### 2.2. Animals

11-week-old male SD rats were purchased from Matt Albert Technology Co. Ltd. (Suzhou, China) and raised in a 12 h light/12 h dark cycle and temperature-controlled room (23°C). All animal experimental procedures were conducted in compliance with the *Guide for the Care and Use of Laboratory Animals* (eighth edition, 2011). The study was approved by the local ethics committee of Suzhou Hospital of Traditional Chinese Medicine. The rats had free access to a standard rodent chow diet (Shuangshi Laboratory Animal Feed Science Co. Ltd., Suzhou, China) and water. The diet contained crude proteins (≥20.5%), water (≤10%), crude fiber (≤5%), crude fat (≥4%), crude ash (≤8%), and a mixture of vitamins and micronutrients. After a week of acclimatization, the rats were randomly divided into two groups, and each group consisted of 7 rats. Vehicle (0.9% NaCl, control) and 50 mg/kg 3DG were given by gastric gavage daily over a two-week period. The rats were fasted overnight before the experiments and euthanized after carotid blood collection. Faeces and intestinal tissue samples were collected and stored at -80°C for further analysis in the meantime. 50 mg/kg 3DG has been chosen in our previous studies [10, 11]. In another experiment, the vehicle group and the 50 mg/kg 3DG group consisting of three rats were also used to determine the alterations in the composition and function of the intestinal microbiota in a 3DG-induced prediabetic rat.

### 2.3. DNA Extraction, PCR Amplification of 16S rRNA Genes, and MiSeq Sequencing

The DNA was extracted from 200 mg samples using the QIAamp DNA Stool Mini Kit (QIAGEN, Hilden, Germany) following the manufacturer's instructions. Faecal samples were running on 1.0% agarose gels to test the concentration and purity of DNA.

PCR amplification of V3-V4 regions in 16S rRNA genes was performed using general bacterial primers 515F (5′-GTGCCAGCMGCCGCGGTAA-3′) and 926R (5′-CCGTCAATTCMTTTGAGTTT-3′). An Illumina MiSeq platform was applied for next-generation sequencing using the PCR products from the amplification of 16S rRNA genes. Sequencing services were provided by Tiny Gene Bio-Tech (Shanghai) Co., Ltd.

### 2.4. Measurements of LPS and GLP-2

The LPS level was determined using commercial Tachypleus amebocyte lysate in accordance with the manufacturer's instructions (Associates of Cape Cod Inc., Beijing, China). GLP-2 concentration was measured by ELISA (Millipore, MA, USA).

### 2.5. Statistical Analyses

Student's *t*-test was applied to evaluate if any differences of LPS, GLP-2, and blood glucose occurred in the two groups. Bacteria taxa with significant differences between the two groups were assessed using Wilcoxon rank-sum tests. Data are presented as the mean ± SD. A *p* value < 0.05 was considered statistically significant. Microbial information statistical analysis and visualization were performed by SPSS version 20, R version 3.6.1, and STAMP version 2.0.6.

## 3. Results

### 3.1. Overall Information of 16S rRNA Gene Sequencing Based on Microbial Species Gene in 3DG-Induced Prediabetic Rats

**Figure 1:**
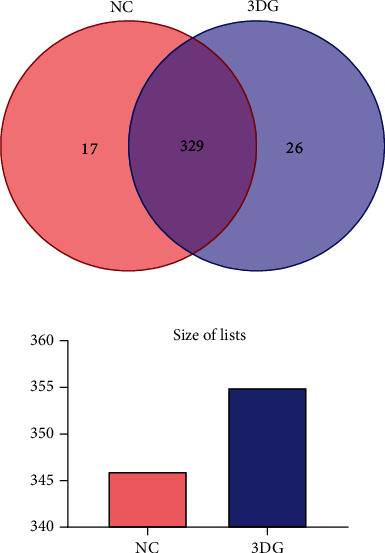
Venn map of OTUs, *n* = 10 for each group. There were 329 common OTUs in NC and 3DG-treated rats. 17 species were specific to the NC group, and 26 species were specific to the 3DG group.

897279 usable optimized raw sequences were obtained, and the average length of which is 456.6 bp. The sequences were clustered at 97% sequence identity into OTUs. 372 OTUs were clustered from 20 samples by bioinformatics statistical analysis, and species classification information of each OTU was obtained. According to the analysis of the Venn map, there were 329 common OTUs in the two groups, and 17 species were specific in the NC group and 26 species were specific in the 3DG group (Figure 1).

### 3.2. Intestinal Microbiota Changes of Bacterial Diversity in 3DG-Induced Prediabetic Rats

**Figure 2:**
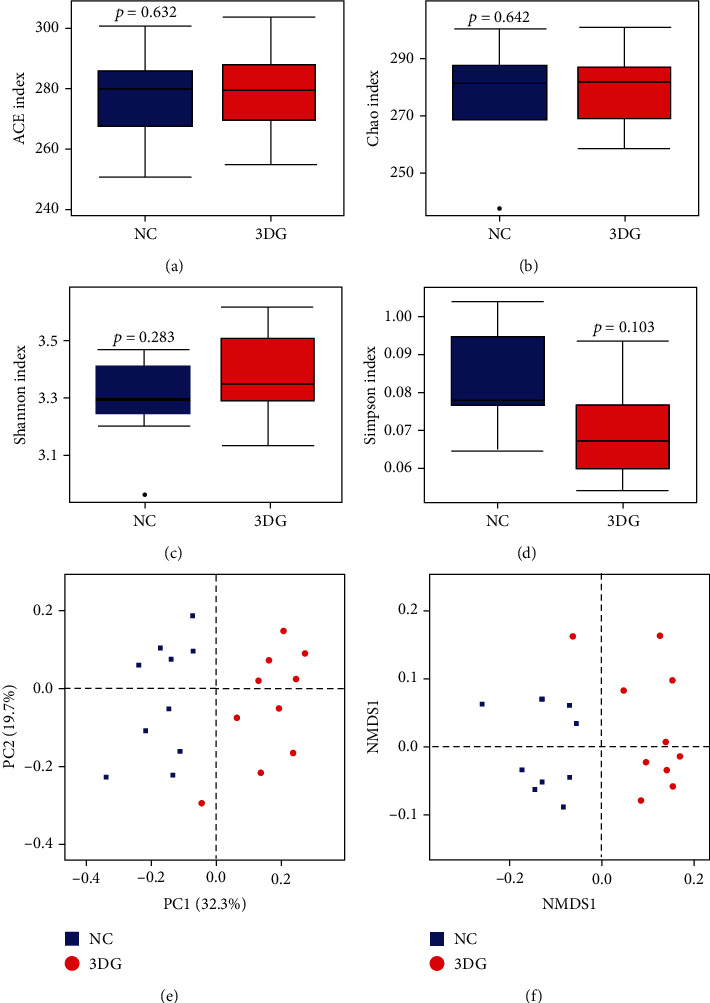
Changes in community diversity of the intestinal microbiota two weeks after intragastric administration of 3DG, *n* = 10 for each group. ACE (a), Chao (b), Shannon (c), and Simpson (d) indices of *α*-diversity were compared between NC and 3DG-treated rats. Statistical testing showed no significant difference for ACE, Chao, Shannon, and Simpson indices. *β*-Diversity analysis including Principal Coordinates Analysis (PCoA) (e) and nonmetric multidimensional scaling (NMDS) (f) performed with Jaccard dissimilarity of the OTU abundance. The contributions of principal coordinate 1 (PC1) is on the *x*-axis and 2 (PC2) is on the *y*-axis.

Effects on richness and diversity of the intestinal microbiota were estimated by the Shannon index, Simpson index, ACE index, and Chao index of *α*-diversity analysis. In the 3DG-treated group, there was an increasing trend in the ACE index (Figure 2(a)), Chao index (Figure 2(b)), and Shannon index (Figure 2(c)). The Simpson index (Figure 2(d)) had a downward trend in the 3DG-treated group compared with the NC group, but there was no statistical significance.

Comparison of the intestinal bacterial community structure between the NC group and the 3DG group was estimated by Principal Coordinates Analysis (PCoA) and nonmetric multidimensional scaling (NMDS) of *β*-diversity analysis based on OTU profiles. PCoA and NMDS were performed with Jaccard dissimilarity of the OTU abundance. As shown in Figures 2(e) and 2(f), the intestinal microbiota community structure of the 3DG group varied from that of the NC group examined in the PCoA and NMDS, which indicated the substantial changes in the overall comparison of the intestinal microbiota.

### 3.3. Alterations in Intestinal Microbiota in 3DG-Induced Prediabetic Rats

Information in the OTU comprehensive classification table is extracted at five levels based on taxonomic information: phylum, class, order, family, and genus, and we identified 7 phyla and 50 genera totally.

**Figure 3:**
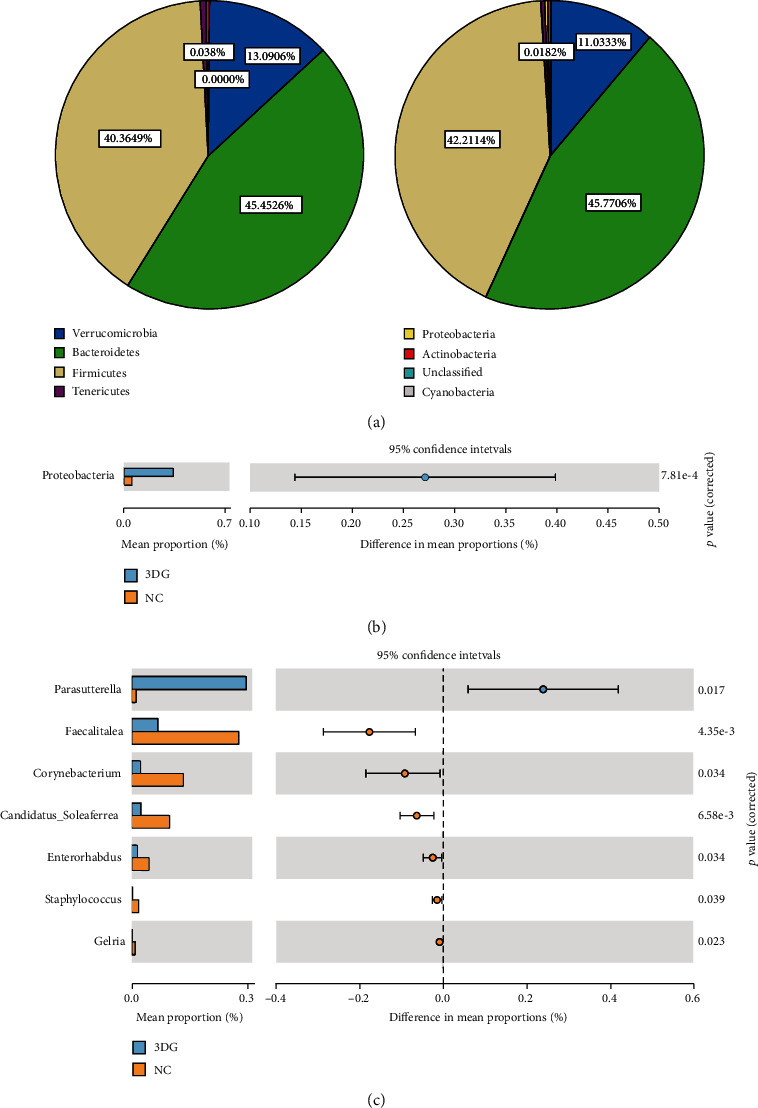
Changes in intestinal microbiota at different bacterial taxonomic information two weeks after intragastric administration of 3DG, *n* = 10 for each group. (a) Pie charts of intestinal microbiota at the phylum level in NC (left) and 3DG-treated (right) rats. (b) Specific difference analysis at the phylum level of the two groups. *Proteobacteria* was significantly increased in the 3DG group (values are the mean ± SD, ^∗^*p* < 0.05). (c) Bar plot of intestinal microbiota differences at the genus level.

At the phylum level, the abundance of dominant bacterial phyla including *Bacteroidetes* (45.5%, 45.8%), *Firmicutes* (40.4%, 42.2%), and *Verrucomicrobia* (13.1%, 11.0%) showed no significant difference between the NC and 3DG groups. Among nondominant bacterial phyla, the abundance of *Tenericutes*, *Actinobacteria*, and *Cyanobacteria* showed no significant difference between the two groups, while the abundance of *Proteobacteria* increased remarkably in the 3DG group (Figures 3(a) and 3(b)).

At the genus level, 3DG-treated rats had an increased abundance of *Parasutterella* and *Caproiciproducens* and decreased abundance of *Candidatus Soleaferrea*, *Enterorhabdus*, *Gelria*, *Aerococcus*, and *Corynebacterium*, compared to the NC group (Figure 3(c)).

**Figure 4:**
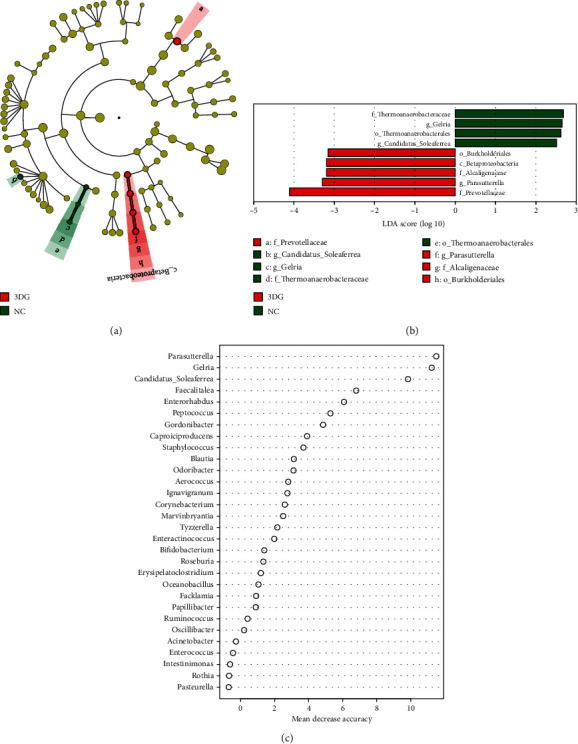
Specific species altered by two-week intragastric administration of 3DG, *n* = 10 for each group. (a) Cladogram using the LEfSe method indicated the phylogenetic distribution of faeces microbes associated with NC (green) and 3DG-treated rats (red). (b) LDA scores (>2) showed the biomarkers and significant bacterial difference between NC and 3DG-treated rats. (c) Bar plot of species importance at the genus level performed by random forest analysis. The abscissa is the importance level, and the ordinate is the species name sorted by importance.

Many microbial taxa significantly differed between the 3DG and NC groups with LDA score > 2 using LEfSe analysis. We found that the bacteria of the *Prevotellaceae* family and *Parasutterela* genus and the bacteria of the *Alcaligencaeae* family and *Burkholderiales* order and its attachment to the *Betaproteobacteria* class were overrepresented in the 3DG group. The bacteria of *Candidatus Soleaferrea* genus, *Gelria* genus, and *Thermoanaerobacteraceae* family and its attachment to *Thermoanaerobacterales* order were apparently more abundant in the NC group (Figures 4(a) and 4(b)).

Random forest analysis was applied to screen out the indicative species. We identified top 30 indicators which differentiated 3DG-treated and NC rats at the genus level (Figure 4(c)). The bacteria of *Parasutterela*, *Gelria*, and *Candidatus Soleaferrea* genera had high importance among species that significantly differed in abundance between the 3DG group and the NC group.

### 3.4. The Impact on Potential Metabolic Functions of Intestinal Microbiota in 3DG-Induced Prediabetic Rats

**Figure 5:**
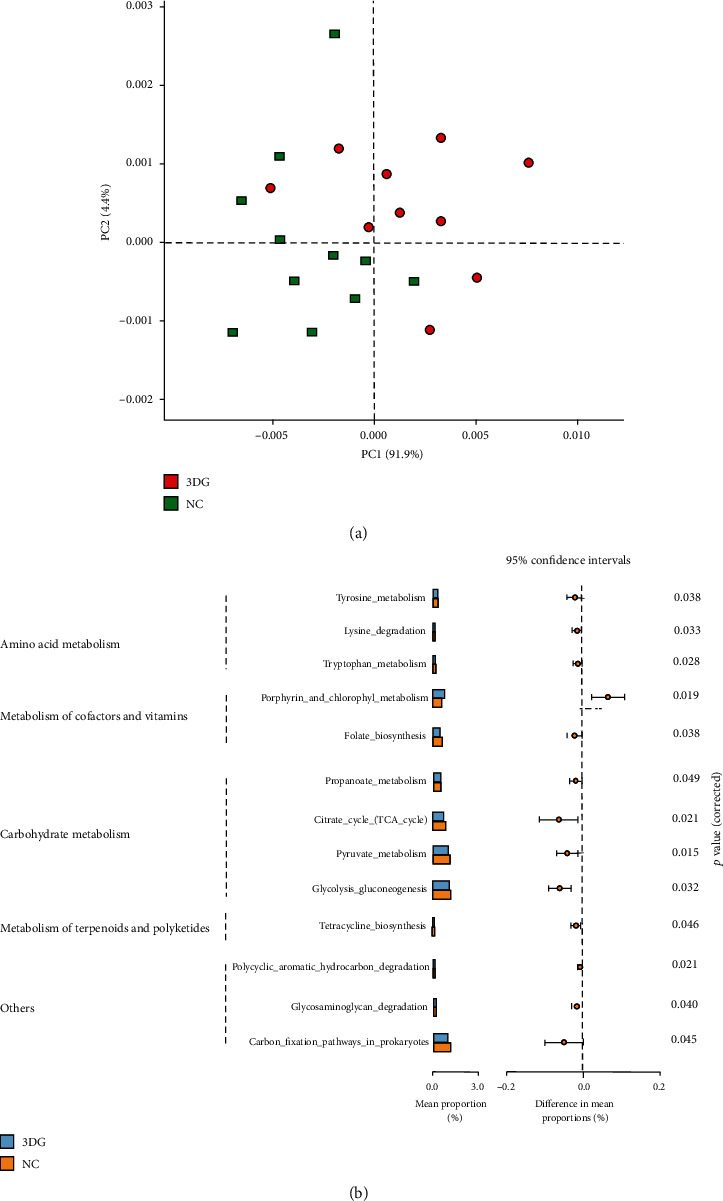
Predictive analysis in the potential metabolic function of intestinal microbiota by PICRUSt, *n* = 10 for each group. (a) PCA of KEGG pathway profile between 3DG-treated and NC rats. (b) The potential metabolism function of KEGG pathways of levels 2 and 3 is shown. A *p* value < 0.05 was considered statistically significant.

PICRUSt was applied to analyse the differences in KEGG pathways of intestinal microbiota between the 3DG and NC groups based on 16S rRNA gene amplicon sequencing profiling. Principal Component Analysis (PCA) showed that the KEGG pathway profile of the intestinal microbiota in 3DG-induced prediabetic rats diverged from that in the NC group (Figure 5(a)).

We found that 45 KEGG pathways were altered after two-week intragastric administration of 3DG. Among these KEGG pathways, 13 KEGG pathways were involved in host metabolic function related to amino acid metabolism, carbohydrate metabolism, metabolism of cofactors and vitamins, and metabolism of terpenoids and polyketides. The abundance of 12 metabolic functions of these KEGG pathways was downregulated, and the abundance of porphyrin and chlorophyll metabolism was upregulated in 3DG-treated rats (Figure 5(b)).

### 3.5. Increased LPS and Decreased GLP-2 Levels in Both Plasma and Intestinal Tissues in 3DG-Induced Prediabetic Rats

**Figure 6:**
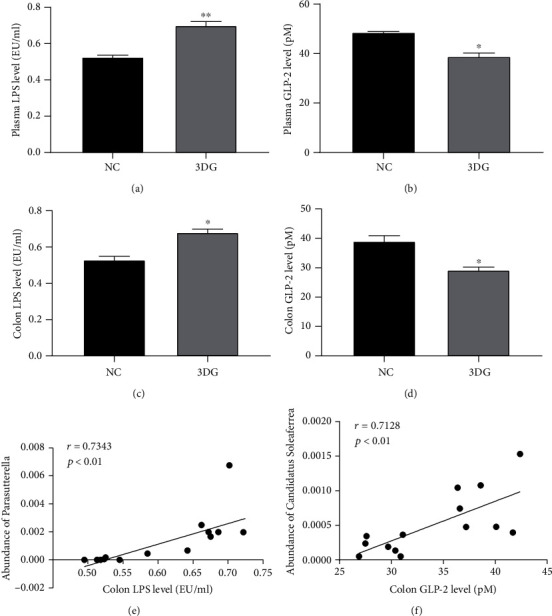
Increased LPS level and decreased GLP-2 level in plasma and colon of rats 2 weeks after intragastric administration of 3DG, *n* = 7 for each group. Plasma (a) and colon LPS (C) levels were significantly increased; plasma (b) and colon (d) GLP-2 levels were significantly decreased compared with the NC group. Values are the mean ± SD. ^∗^*p* < 0.05, ^∗∗^*p* < 0.01. (e) Correlation between colon LPS level and abundance of *Parasutterella*. (f) Correlation between colon GLP-2 level and abundance of *Candidatus Soleaferrea*. The inset corresponds to Pearson's *r* correlation and corresponding *p* value.

Our previous study has shown that two-week oral administration of 3DG impaired gut permeability in rats [15]. Since glucagon-like peptide-2 (GLP-2) and LPS play an important role in maintaining gut permeability, we observed the levels of GLP-2 and LPS in plasma and intestinal tissues of rats following a two-week administration of 3DG by gastric gavage. As shown in Figures 6(a)–6(d), in 3DG-treated rats, LPS levels in both plasma and colon were significantly increased, and GLP-2 concentrations in both the plasma and colon were significantly decreased. Furthermore, the correlational relationship between the intestinal microbiota and LPS/GLP-2 was determined. We found that colon LPS levels were positively correlated with abundance of *Parasutterella* (Figure 6(e)). Colon GLP-2 levels were positively correlated with abundance of *Candidatus Soleaferrea* (Figure 6(f)).

### 3.6. Two-Week Oral Administration of 3DG Induced the Impaired Glucose Regulation in Rats

**Figure 7:**
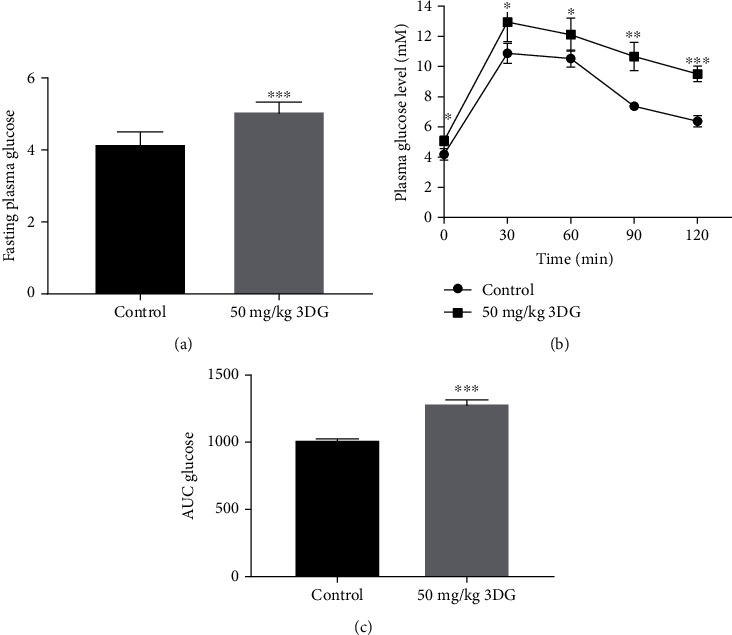
Intragastric administration of 3DG for 2 weeks caused normal rats to develop increased fasting blood glucose concentration and impaired oral glucose tolerance. *n* = 7 for each group. (a) Fasting plasma glucose levels were measured in rats after 2 weeks of 3DG (50 mg/kg) or vehicle treatment. (b) OGTT (2.5 g/kg) was performed after 2-week administration of 3DG (50 mg/kg) or vehicle in rats. (c) The glycaemic response was expressed as the area under the curve. Values are the mean ± SD. ^∗^*p* < 0.05, ^∗∗^*p* < 0.01, and ^∗∗∗^*p* < 0.001 compared with the control group.

As shown in Figure 7(a), the level of fasting blood glucose in rats treated with 50 mg/kg 3DG was significantly higher than that in the control group. Compared with the control group, the group of 3DG-treated rats had impaired oral glucose tolerance (Figure 7(b)). The glucose area under the curve (AUC) in the 3DG-treated group was significantly higher than that of the control group (Figure 7(c)).

## 4. Discussion

Our recent results from animal studies and clinical research have revealed that 3DG is an independent factor associated with the development of prediabetes. In this study, we comprehensively examined the diversity, composition, and function of the intestinal microbiota in 3DG-induced prediabetic rats. 3DG-induced prediabetic rats (the impaired fasting glucose and oral glucose tolerance) displayed the alterations in *β*-diversity of intestinal microbiota, the increased abundance of *Parasutterella* and *Caproiciproducens*, and the decreased abundance of *Candidatus Soleaferrea*, *Enterorhabdus*, *Gelria*, *Aerococcus*, and *Corynebacterium*. 13 KEGG pathways related to host metabolic functions were changed in 3DG-induced prediabetic rats.

Research found that there was no significant difference in *α*-diversity of prediabetes and T2D patients [22]. Meanwhile, there was a significant difference in intestinal microflora between diabetic and normal people, and the decrease of probiotics such as intestinal *Lactobacillus* and *Bifidobacterium* is closely associated with the abnormal glucose tolerance in T2D patients [23]. Our results of *α*-diversity analysis indicated that community diversity including richness and evenness showed considerably stable two weeks after the intragastric administration of 3DG. Interestingly, differentiated OTUs suggested that there were significant differences in specific OTUs of 3DG-induced prediabetes. Results of *β*-diversity analysis based on OTU profiles also indicated the substantial alterations in the overall composition of 3DG-induced prediabetes.

Intestinal microbiota plays an important role in regulating and maintaining the integrity of the intestinal mucosal barrier. The balance of commensal bacteria and probiotics can promote the integrity of gut barriers [24, 25]. LPS components in the bacterial cell wall have a kind of pathogen-associated molecular pattern and can induce destruction of the intestinal epithelial barrier, increased production of inflammatory mediators, and excessive tissue damage by reducing the expression of tight junction proteins [26]. Classically, GLP-2, secreted by the metabolites (short-chain fatty acids) of intestinal microbiota, is considered a trophic hormone involved in maintaining intestinal epithelial morphology and function. Our previous study has shown that two-week oral administration of 3DG impaired gut permeability in rats [15]. In this study, we further observed the increased LPS levels and decreased GLP-2 levels in 3DG-induced prediabetic rats. Meanwhile, the increased abundance of *Parasutterella* which is positively correlated with LPS and decreased abundance of *Candidatus Soleaferrea* which is positively correlated with GLP-2 genus were observed. The bacteria of the *Parasutterella* genus were shown to positively correlate with the chronic inflammatory state and drove insulin resistance [27]. The abundance of *Parasutterella* remarkably increased in HFD-induced T2D mice and decreased after the treatment of novel antidiabetic monomers [28]. The *Candidatus Soleaferrea* genus was known to possess anti-inflammatory effects by secreting metabolites and intestinal homeostasis protection properties [29, 30]. Our findings provide a possible mechanism linking exogenous 3DG intake to the increased gut permeability.

The bacteria of *Prevotellaceae* were reported to have a positive correlation with the AMPK signal pathway and improve lipid metabolism disorder in ob/ob mice [31]. However, another study pointed out that the abundance of *Prevotellaceae* increased significantly in T2D [32]. In agreement, we found that the bacteria of the *Prevotellaceae* family were overrepresented in 3DG-induced prediabetic rats.

*Caproiciproducens*, *Enterorhabdus*, *Aerococcus*, and *Corynebacterium*, especially for the potential biomarker *Gelria* genus, showed significant changes in the current study. As far as we are concerned, these altered bacteria were not found to be associated with metabolic diseases and deserved to be further investigated.

The development of T2D and prediabetes is associated with dynamic changes in host metabolism. Accumulating evidence has suggested that intestinal microbiota plays a vital role in host metabolism [33]. Recently, PICRUSt is commonly used to predict the functional composition of microbiota [34]. In the current study, PICRUSt analysis showed that 3DG caused the downregulation of carbohydrate metabolism and amino acid metabolism, suggesting that metabolic function is inhibited in 3DG-induced prediabetic rats. Metabolism of cofactors and vitamin disorders such as the downregulation of folate metabolism (a kind of diabetes biomarker) increased the risk of metabolic diseases [35]. Previous research found a decrease in functions involving cofactor and vitamin metabolism and amino acid metabolism in metabolic diseases such as T2D and obesity [36, 37]. The results from PICRUSt analysis also further supported our previous findings that provide a possible mechanism linking exogenous 3DG intake to the development of prediabetes.

## 5. Conclusions

The alterations in composition and function of the intestinal microbiota were observed in 3DG-treated rats, which provides a possible mechanism linking exogenous 3DG intake to the development of prediabetes. Combining with our previous study, our findings further suggested that the reduction of dietary 3DG intake contributes to the prevention and treatment of diabetes. To further verify the involvement of intestinal microbiota in the 3DG-induced prediabetes, the faeces microbiota of the 3DG-induced diabetes rats will be transplanted to germ-free or SPF mice.

## Data Availability

The datasets used and analysed during the current study are available from the corresponding authors on reasonable request.
